# Mechanisms and treatments of methamphetamine and HIV-1 co-induced neurotoxicity: a systematic review

**DOI:** 10.3389/fimmu.2024.1423263

**Published:** 2024-08-19

**Authors:** Lin Miao, Haowei Wang, Yi Li, Jian Huang, Chan Wang, Hanxin Teng, Lisha Xu, Xue Yang, Yunqing Tian, Genmeng Yang, Juan Li, Xiaofeng Zeng

**Affiliations:** ^1^ NHC Key Laboratory of Drug Addiction Medicine, School of Forensic Medicine, Kunming Medical University, Kunming, China; ^2^ Department of Pathogen Biology and Immunology, School of Basic Medical Science, Kunming Medical University, Kunming, China

**Keywords:** methamphetamine, psychostimulant, HIV-1, neurotoxicity, programmed cell death, blood-brain barrier, sigma 1 receptor

## Abstract

Combination antiretroviral therapy (cART) has dramatically reduced mortality in people with human immunodeficiency virus (HIV), but it does not completely eradicate the virus from the brain. Patients with long-term HIV-1 infection often show neurocognitive impairment, which severely affects the quality of life of those infected. Methamphetamine (METH) users are at a significantly higher risk of contracting HIV-1 through behaviors such as engaging in high-risk sex or sharing needles, which can lead to transmission of the virus. In addition, HIV-1-infected individuals who abuse METH exhibit higher viral loads and more severe cognitive dysfunction, suggesting that METH exacerbates the neurotoxicity associated with HIV-1. Therefore, this review focuses on various mechanisms underlying METH and HIV-1 infection co-induced neurotoxicity and existing interventions targeting the sigma 1 receptor, dopamine transporter protein, and other relevant targets are explored. The findings of this review are envisaged to systematically establish a theoretical framework for METH abuse and HIV-1 infection co-induced neurotoxicity, and to suggest novel clinical treatment targets.

## 1 Introduction

Acquired immunodeficiency syndrome (AIDS) is a highly infectious disease caused by infection with the human immunodeficiency virus (HIV). Most people with AIDS are infected with HIV-1, which is the most prevalent and more virulent subtype. It has been demonstrated that HIV-1 may trigger HIV-associated neurocognitive disorder (HAND) by inflicting damage to the neurovascular units (NVU), blood-brain barrier (BBB) dysfunction, and inflammatory response (1). It has been shown that the interplay between diverse HIV-1 viral proteins (Tat, Vpr, and gp120) and neural cells may be linked to the pathogenesis of HIV-1-induced neurotoxicity, which results in cellular injury and anomalous alterations in the CNS (2). However, the precise mechanism of action is not yet well defined. Despite a significant reduction in mortality rates among HIV-1 patients due to combination antiretroviral therapy (cART), chronic viral infections continue to persist in the brain (3). Moreover, it has been shown that long-term cART may disrupt mitochondrial function, affect metabolism, and even cause neurotoxicity and fatal complications (4, 5). Therefore, we need to explore safer and more effective ways for HIV treatment.

The global issue of substances misuse significantly affects the social welfare and public health. The World Drug Report 2024 by the United Nations Office on Drugs and Crime (UNODC) indicates that 13.9 million individuals engaged in substances injections in 2022, and about one in eight injecting substances abusers will be living with HIV-1 (6). Methamphetamine (METH), a highly addictive psychostimulant, is widely abused and can lead to severe health risks following withdrawal (7, 8). Prolonged METH use has also been reported to negatively impact memory, learning, and cognitive function, leading to symptoms such as paranoia, insomnia, irritability, hallucinations, and delusions (9). In severe cases, METH abuse may contribute to the development of neurodegenerative disorders such as Alzheimer’s disease (AD) and Parkinson’s disease (PD). And yet, there are currently no the US Food and Drug Administration (FDA) -approved drugs for the treatment of METH-induced neurotoxicity (10).

METH abuse is often associated with HIV-1 infection through needle sharing and high-risk sexual activity among users. HIV-1-infected individuals who abuse METH exhibit higher viral loads and more severe cognitive dysfunction (11). Concurrently, studies have shown that the pathways of neurotoxicity can be induced by METH and HIV are somewhat similar. For instance, they can cause mitochondrial dysfunction and altered metabolic pathways (12–14), which in turn can lead to programmed cell death and neuroinflammatory (15–17). However, the specific mechanisms underlying this synergistic neurotoxicity are not fully understood and effective treatments are lacking. Therefore, this paper reviews the mechanisms of synergistically induced neurotoxicity by METH and HIV-1 and proposes potential therapeutic targets.

## 2 Potential mechanisms of neurotoxicity co-induced by METH and HIV-1

### 2.1 Oxidative stress and endoplasmic reticulum stress

Oxidative stress, a harmful result of free oxygen radicals in the body, is commonly associated with the processes of ageing and disease (18). Endoplasmic reticulum stress (ERS) is characterized by an atypical accumulation of unfolded or misfolded proteins, resulting from an increased demand for properly folded proteins in response to external stimuli, ultimately leading to ER dysfunction (19). The interaction between ERS and oxidative stress was apparent. The disruption of redox homeostasis in the endoplasmic reticulum caused by oxidative stress has been shown to significantly affect endoplasmic reticulum function, leading to endoplasmic reticulum signaling activation, which could result in ERS development and could produce high levels of reactive oxygen species (ROS), thereby exacerbating oxidative stress (20).

METH has been demonstrated to damage dopaminergic neurons through oxidative stress processes. Upon entering the neuron, METH displaces dopamine (DA) from vesicles, leading to an increase in intracellular and synaptic gap DA levels, which results in elevated auto-oxidation and DA metabolism, leading to higher ROS production (21). The resulting damage to cellular proteins, lipids, and DNA leads to a loss of cellular function and subsequent neurotoxicity, which is further exacerbated when METH and HIV-1 act together (22). Within the human innate immune macrophage cell line system, METH induces significant cellular ROS production, thereby activating the expression of interleukine-1β (IL-1β) and tumor necrosis factor-alpha (TNF-α) in a dose-dependent manner. Additionally, METH-induced oxidative stress is further exacerbated by the presence of HIV-1 Tat protein (23). Combined exposure to METH and HIV-1 synergistically induces oxidative stress by activating transient receptor potential melastatin 2 (TRPM2) channels in endothelial cells, thereby resulting in ROS production, which further exacerbates the toxic effects on the nervous system by compromising the antioxidant defenses of catalase (CAT), glutathione peroxidase (GSH-Px), and superoxide dismutase (SOD) (1). TRPM2 channels can mediate oxidative stress by activating NLRP3 inflammasome and microglia or inducing TNF-α production, triggering autophagy in human cerebrovascular pericytes and damaging BBB (24).

It was also found that METH caused a significant increase in ERS-related proteins Bip, ATF-6, ATF-4, eIF2α, and CHOP in C57BL/6 mice, where memory loss and cognitive impairment were improved when treated with taurine, an ERS inhibitor, suggesting that METH-induced memory loss was associated with ERS (25).Abnormal mitochondrial function is a significant contributor to oxidative stress (26). Sirtuins (SIRTs) are NAD^+^-dependent deacetylases involved in mitochondrial biogenesis, protein responses, and intrinsic apoptosis. The activation of SIRTs has been reported to exert a neuroprotective effect in conditions such as AD and PD (27). In HIV-1 disease models, the miR-505/SIRT3 axis was implicated in mitochondrial oxidative stress, associated with the induction of the HIV-1 Tat protein-mediated microglia senescence phenotype, leading to elevated superoxide production within mitochondria (28). Consequently, HIV-1 Tat protein may induce oxidative stress and ERS by inhibiting SIRT1 and SIRT3, thereby diminishing their neuroprotective properties (28, 29). Mitochondria-associated endoplasmic reticulum membranes (MAMs) represent direct contact sites between the ER and mitochondria, functioning as critical platforms for the coordination of essential cellular processes, including mitochondrial dynamics and calcium homeostasis (30). Notably, the strategic targeting of MAMs to modulate astrocyte mitochondrial function holds potential as a promising approach to enhance the metabolic and antioxidant coupling between astrocytes and neurons, thereby promoting neuronal resilience against CNS pathologies (30). It has also been shown that METH and HIV-1-induced astrocyte excitatory amino acid transporter-2 dysfunction can be restored by maintaining calcium homeostasis (31).

**Figure 1:**
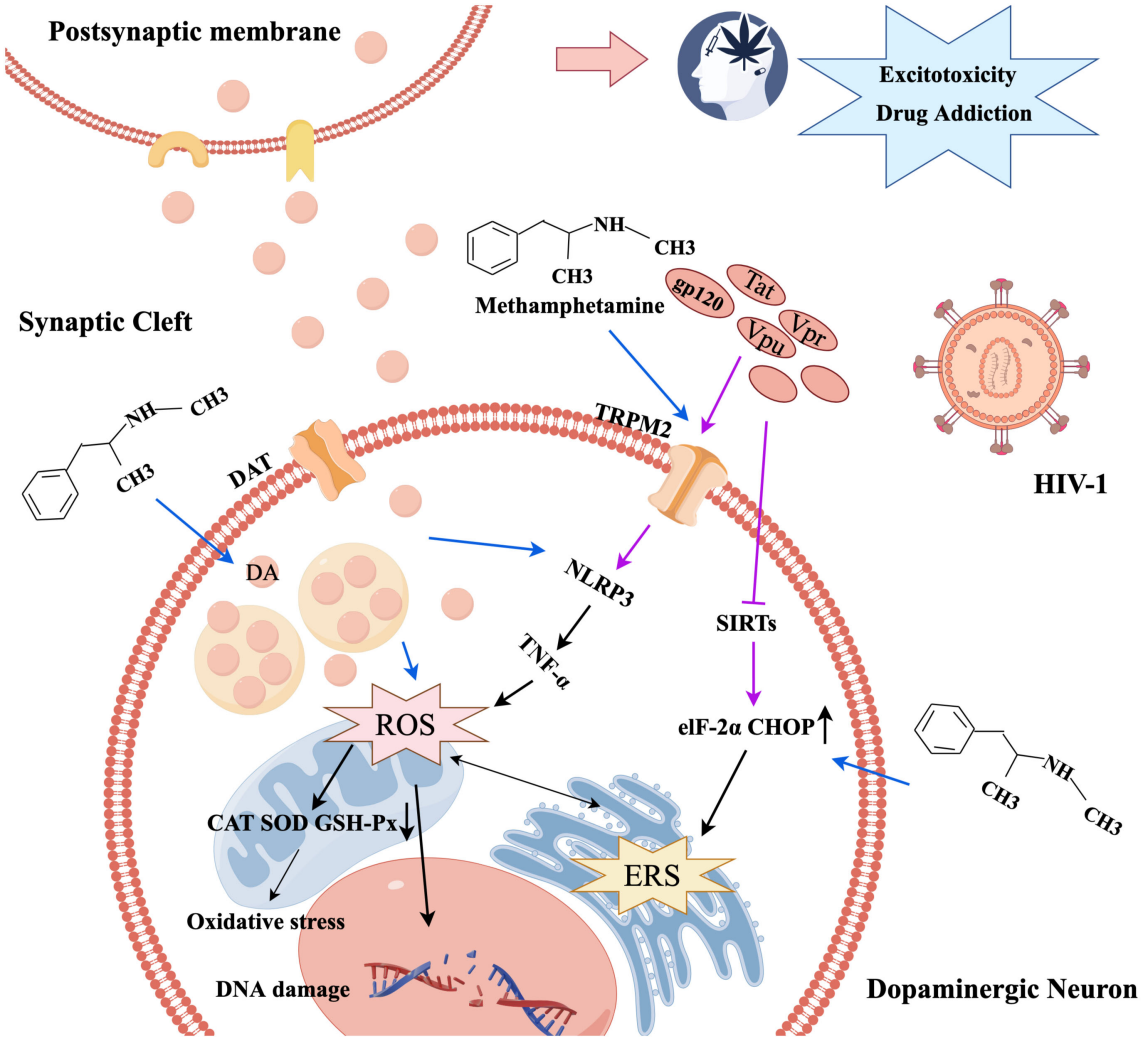
The mechanism diagram of oxidative stress and endoplasmic reticulum stress caused by METH and HIV-1.

Regulation of endoplasmic reticulum-related signaling pathways is critical for cellular homeostasis (19). Results of a study evaluating the effects of 7-day HIV-1 infection and chronic METH exposure on the endoplasmic reticulum and mitochondria showed that the expression level of ERS-related inositol-requiring protein 1α (IRE1α) was significantly upregulated (32). These results imply that ERS is implicated in the synergistic effect of HIV-1 and METH on the induction of neurocognitive impairment. Nonetheless, moderate ERS is beneficial for the body, but excessive ERS can cause neurocognitive dysfunction; thus, both oxidative stress and ERS are important factors in neurotoxicity caused by METH and HIV-1 (**Figure 1**).

### 2.2 Programmed cell death

**Figure 2:**
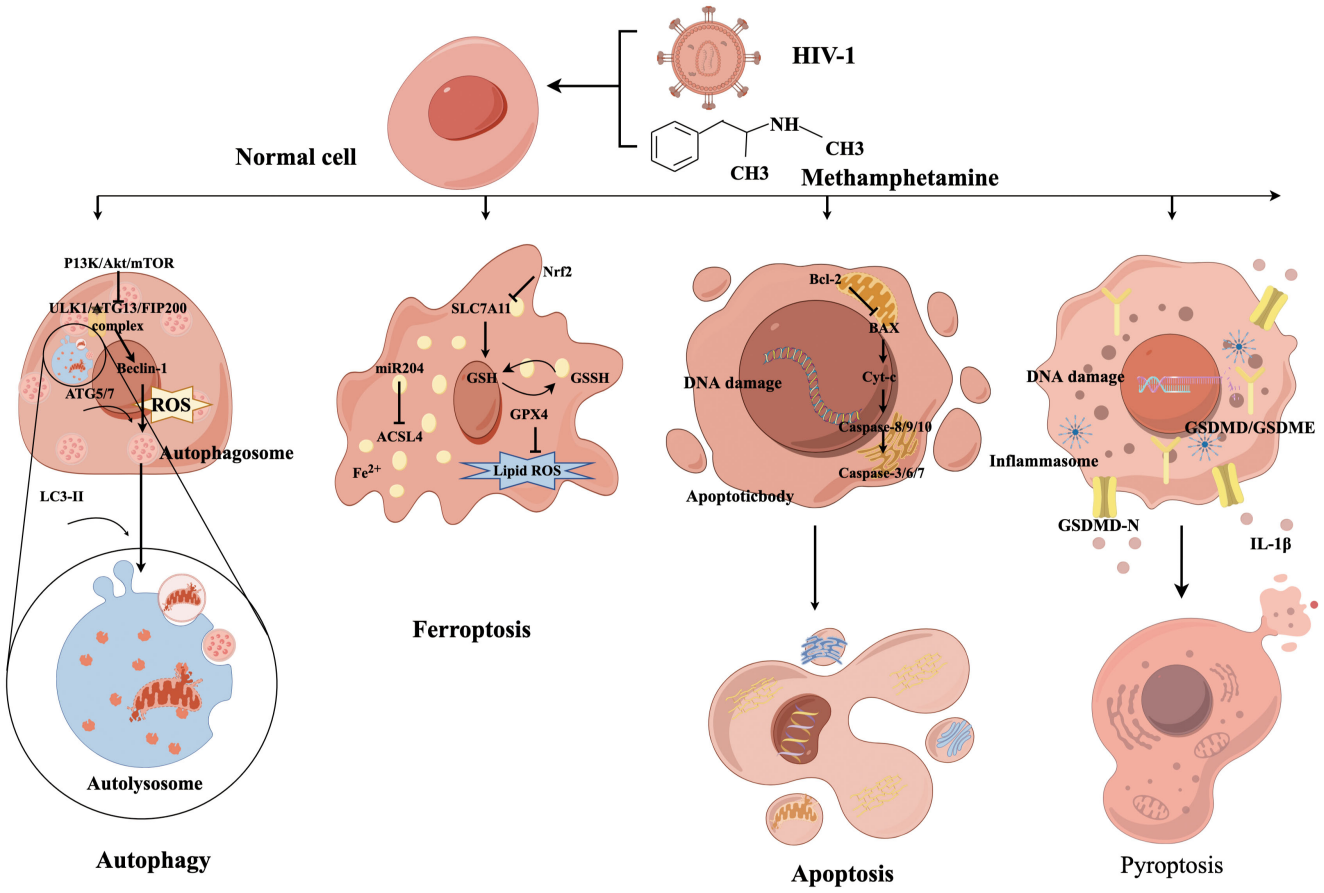
The mechanism diagram of programmed cell death caused by METH and HIV-1.

Presently, the prevalent types of programmed cell death include apoptosis, pyroptosis, autophagy, necrosis, and ferroptosis (33). The molecular mechanisms underlying these forms of cell death are not self-contained, and recent findings indicate an intricate interplay between them, collectively promoting nerve cell death (**Figure 2**) (34).

#### 2.2.1 Autophagy

Autophagy is a crucial physiological mechanism for preserving cellular homeostasis and energy conservation. This process is primarily initiated by the activation of ULK1, Atg13, and FIP200 complexes via the PI3K/Akt/mTOR pathway, which then triggers the commencement of autophagic program (35). The acceleration of cell death resulting from dysregulation of autophagy occurs when the body is overstimulated. Autophagy dysfunction has been identified as a contributing factor to mental disorders and synaptic damage in degenerative diseases (36). Meanwhile, autophagy is the primary pathway for the degradation of long-lived proteins and organelles and is closely associated with survival, death, and neuron transformation (33). The formation of double-membrane vesicles, known as autophagosomes, is facilitated by the copolymerization of a series of Atg complexes that can capture organelle fragments in the cytoplasm (37). The completion of autophagy depends on the correct formation, maturation, and fusion with lysosomes of autophagosomes.

The specific role of autophagy in the body is twofold. On the one hand, the potential for the accumulation of misfolded proteins following HIV-1 infection to induce ERS, autophagy can effectively remove these toxic proteins, thereby reducing ERS and inhibiting the progression of HAND. On the other hand, an autophagic imbalance may result in the build-up of harmful substances in neurons (38). Similarly, METH enhanced HIV-1 gp120-mediated autophagy via Beclin-1 and Atg5/Atg7, thereby exerting a protective effect on astrocytes (39). Recent research on the gut-brain axis has revealed that the METH-abused BALB/c mouse model with continuously increasing multiple doses can induce neurotoxicity and enteritis and alter the gut microbiota and fecal metabolites, which is closely associated with autophagy (40). Our previous study revealed that HIV-1 Tat protein can promote autophagy in nerve cells in a concentration-dependent manner, and the induction of autophagy by HIV-1 Tat protein was found to decrease cellular activity, signifying its detrimental impact on the nervous system (41).

METH abuse has been observed to result in elevated systemic inflammation and CNS impairment in HIV-1-infected individuals. METH was found to inhibit macrophage phagocytosis of aggregated amyloid-β, increase total ROS, and dysregulate the autophagic process in HIV-1-infected human macrophages (42). Furthermore, previous research demonstrated that the combined effects of HIV-1 Tat protein and METH can induce autophagy in primary tree shrew midbrain neuronal cells through mTOR signaling and the Atg5/Atg7 pathway (43). The combined impact of METH and HIV-1 Tat proteins results in a significant increase in LC3-II expression and a blockade of autophagic flow in primary human neuronal cells, resulting in increased mitochondrial DRP1-dependent breakdown or rupture and neuronal degenerative changes by inhibiting mitochondrial autophagy (44). These findings suggest that the combined action of METH and HIV-1 Tat proteins induces autophagy, thereby contributing to neurotoxic damage.

#### 2.2.2 Ferroptosis

Ferroptosis is a recently discovered iron-dependent form of programmed cell death distinct from apoptosis, necrosis, and autophagy (45). Although Ferroptosis was initially proposed in 2012, prior research has identified irregularities in iron metabolism within the CNS (46). Ferroptosis is characterized by unique morphological and biochemical features, including depletion of intracellular GSH and inactivation of GSH-Px4, resulting in the accumulation of lipid peroxidation (34). Ferroptosis induction is closely associated with oxidative stress, a phenomenon that METH contributes to through various pathways, ultimately affecting neurocognitive function. Many studies have implied the importance of maintaining normal intracellular iron levels for proper development and functioning of the CNS. Consequently, Ferroptosis may be pivotal in METH-induced neurotoxicity.

Several studies have established a correlation between ferroptosis and various neurodegenerative disorders such as PD, AD, and HAND (47). Neurons and glial cells exhibit elevated susceptibility to ferroptosis and neurodegeneration, with microglia being the most iron-laden cells. Iron-activated microglia may participate in the aberrant elimination of neurons and synapses, thereby exacerbating ferroptosis-induced neurodegeneration (48). Researchers found, as early as the 1990s, that ferritin concentrations increased, and iron-mediated oxidative stress was evident as AIDS progressed (49). According to a recent clinical study, elevated concentrations of iron transport proteins in the cerebrospinal fluid are associated with the incidence of HAND (50). The underlying mechanism of HAND may be attributed to HIV-1-related proteins inducing ferroptosis, where HIV-1 Tat protein specifically triggers ferroptosis in microglia by suppressing miR-204 expression, which subsequently upregulates ACSL4 expression, leading to increased lipid peroxidation. This process ultimately results in microglial activation and secretion of proinflammatory cytokines, culminating in neuroinflammation and neurotoxicity (48).

SLC7A11 is a crucial cystine/glutamate transporter protein constituent that expedites GSH synthesis and impedes ferroptosis (51). Our research team discovered that METH and HIV-1 Tat proteins can collaboratively instigate ferroptosis in BV2 cells, which is antagonized by transcription factor NF-E2-related factor (Nrf2) by regulating SLC7A11, thus providing a theoretical basis for investigate the synergistic effects of METH and HIV-1 induced neurotoxicity (17). However, the precise mechanisms underlying ferroptosis are poorly understood and warrant further investigation.

#### 2.2.3 Apoptosis

Apoptosis is among the earliest identified forms of programmed cell death mechanisms (52). Unlike necrosis, apoptosis is an activecell death process that does not involve rupture of the cell membrane. It is often characterized by cell shrinkage, nuclear breakage, budding of the cytoplasmic membrane, and formation of apoptotic vesicles (53). Apoptosis has been a research hotspot for METH and HIV-1 induced neurotoxicity.

Apoptosis can be divided into an initiation phase and an execution phase, where the former is generally mediated by caspases 8, 9, and 10 and the latter by caspases 3, 6, and 7 (54). Among them, caspases 8 and 10 are initiators of exogenous apoptosis, and caspase 9 is an initiator of endogenous apoptosis. The activation of caspase-3 heralds the beginning of the apoptotic process and is considered a key enzyme that leads to apoptosis. A variety of proteins play equally important roles in the development of apoptosis. Bcl-2 and Bcl-XL act as potent apoptosis inhibitors to prevent apoptosis. Factors such as Bak, Bad and Bax promote apoptosis. In addition, P53 and Cyt-c are also involved in apoptosis development (55).

As previously mentioned, autophagy may have deleterious effects, resulting in autophagic cell death, and multiple intermediates of programmed cell death engage in intricate interactions (56). In an *in vitro* astrocyte experiment, the combined effects of METH and gp120 were observed, where inhibition of autophagy resulted in significant astrocyte apoptosis, indicating that autophagy plays a protective role against apoptosis caused by METH and gp120 (39). Similarly, another study found that gp120 and METH-induced oxidative stress through the cytochrome (CYP) P450 and NADPH oxidase (NOX) pathways result in astrocyte apoptosis (57), implying that inhibiting these two pathways could significantly help suppress the apoptosis of astrocytes. These findings demonstrated that METH and gp120 can induce apoptosis in several ways. In addition to gp120, a significant increase in TUNEL-staining-positive cells was also found in the hCMEC/D3 model of human microvascular endothelial cells following the combined action of HIV-1 Tat protein and METH (24). This indicates that both METH and multiple viral proteins of HIV-1 can induce apoptosis and that efficacious interventions are currently unavailable.

#### 2.2.4 Pyroptosis

Pyroptosis is a mode of inflammatory cell death activated through two main pathways: gasdermin D (GSDMD)-dependent activation regulated by caspase-1/4/5/11 and GSDME-dependent activation regulated by caspase 3 (58). Activated caspases cleave the hinge region between the N- and C-terminal domains of GSDMD or GSDME to recognize and bind to phospholipid molecules in the cell membrane, This results in the formation of pores or perforations within the cell membrane, leading to alterations in cellular osmolarity and ultimately cell membrane rupture and cell death (59).

Substances misuse has been shown to accelerate neurological symptoms in HIV-1-infected patients by facilitating HIV-1 entry into the brain and triggering an immune response, thereby mediating neuroinflammation and pyroptosis through the activation of microglia and the release of neurotoxins (60). Studies have disclosed that METH can induce GSDME-dependent cell death of hippocampal neurons through the ERS pathway (61). In addition, manifestations of cognitive impairment and changes in the NLRP1/Caspase1/GSDMD signaling pathway were found in an *in vivo* model of METH administration in rats (62). The use of aspirin and NLRP1 siRNA significantly attenuated METH-induced cognitive deficits while decreasing the activities of NLRP1 and cleaved caspase-1, IL-1β, and TNF-α, providing further evidence of complex programmed cell death mechanisms. Similarly, one study reported HIV-1 gp120-induced NLRP3-dependent pyroptosis and IL-1β production in microglia (63). Long-term administration of MCC950 (an NLRP3 inhibitor) to gp120 transgenic mice attenuated neuroinflammation and neuronal death, promoted neuronal regeneration, and restored impaired neurocognitive functions (64).

All the aforementioned findings suggest a link between METH and HIV-1 and pyroptosis. Indeed, a recent study showed that METH enhanced HIV-1 gp120-induced activation of microglia and NLRP3 inflammasome, while elevating GSDMD and GSDMD-N expression (65). Thus, activation of microglia-mediated neuroinflammation and pyroptosis might be one of the HAND etiological factors. Nevertheless, the investigation into METH and HIV-1 acting synergistically in inducing pyroptosis is still in its infancy. Further research is required to elucidate the interrelationships between pyroptosis and other mechanisms.

### 2.3 Neuroinflammation

Neuroinflammation is an important mechanism of neurotoxicity that has been closely associated with the activation of inflammasomes, glial cells, and cytokines (9, 66–68). The activation of microglia and astrocytes is a crucial component of the synergistic induction of neuroinflammation by METH and HIV-1. It has been shown that METH can activate inflammatory cells such as astrocytes and microglia, leading to the secretion of TNF-α and IL-1β inflammatory factors (23). Upon HIV-1 infection of human microglia, pro-IL-1β is secreted at 4 h, and mature IL-1β is secreted at 24 h, accompanied by caspase-1 activation, initiating the inflammatory process (69). Furthermore, HIV-1 infection induces a notable release of proinflammatory cytokines, namely IL-1β and IL-18. Exposure of monocytes to HIV-1 Tat has been observed to cause IL-1β release, which can lead to neuronal cell death and HAND through a direct or indirect proinflammatory mechanism (70). Similarly, the severity of this inflammatory response is elevated in HIV-1 patients with a history of METH abuse (71).

At present, five primary types of inflammasomes, namely NLRP3, NLRP1, NLRC4, IPAF, and AIM2, have been extensively studied and are known to play crucial roles in the onset and progression of various diseases (72). In a study involving METH abuse in rats, elevated NLRP1 activity was observed in the hippocampus, and NLRP1 inhibition significantly ameliorated METH-induced cognitive impairment (62). Moreover, inhibition of the NLRP3 inflammasome has the potential to mitigate motor impairments and cerebellar alterations induced by METH administration (68). Similarly, HIV-1 activates inflammasomes through diverse mechanisms, resulting in immune cell death and associated pathological changes (73). This shows a notable correlation between inflammasomes and neuroinflammation induced by METH and HIV-1.

The findings of our study indicated that microglia in the prefrontal cortex of METH abusers with concurrent HIV-1 infections exhibited significant activation (74). Additionally, METH and HIV-1 led to the activation of NLRP3 inflammasomes and caused neuroinflammation, as well as neuronal apoptosis in the prefrontal cortex and hippocampus (63). These results have suggested that neuroinflammation in HIV-1-infected individuals with METH abuse may contribute to neuronal apoptosis and consequent damage to the nervous system. Neuroinflammation involves a multifaceted interplay of mechanisms, including apoptosis and oxidative stress (75). Therefore, investigating the interplay between neuroinflammation and other mechanisms is a promising area for future research.

### 2.4 Blood-brain barrier damage

The BBB serves as a critical interface between the brain and the peripheral circulation and plays a vital role in maintaining homeostasis within the CNS (76). The neurovascular unit (NVU), which comprises endothelial cells, pericytes, astrocytes, microglia, neurons, and basement membranes, is the main functional unit of the blood-brain barrier (77). To access the CNS, both METH and HIV-1 must traverse the BBB. It has been established that the benzene ring and methyl group in the chemical structure of METH render it lipid-soluble and capable of traversing cell membranes and even the BBB, thereby exerting an effect on the central nervous system. HIV-1 may gain access to the central nervous system by infecting endothelial cells or mononuclear macrophages via a “Trojan horse” mechanism (78). Notably, HIV-1 infection and METH abuse synergistically impairs the BBB (79).

**Figure 3:**
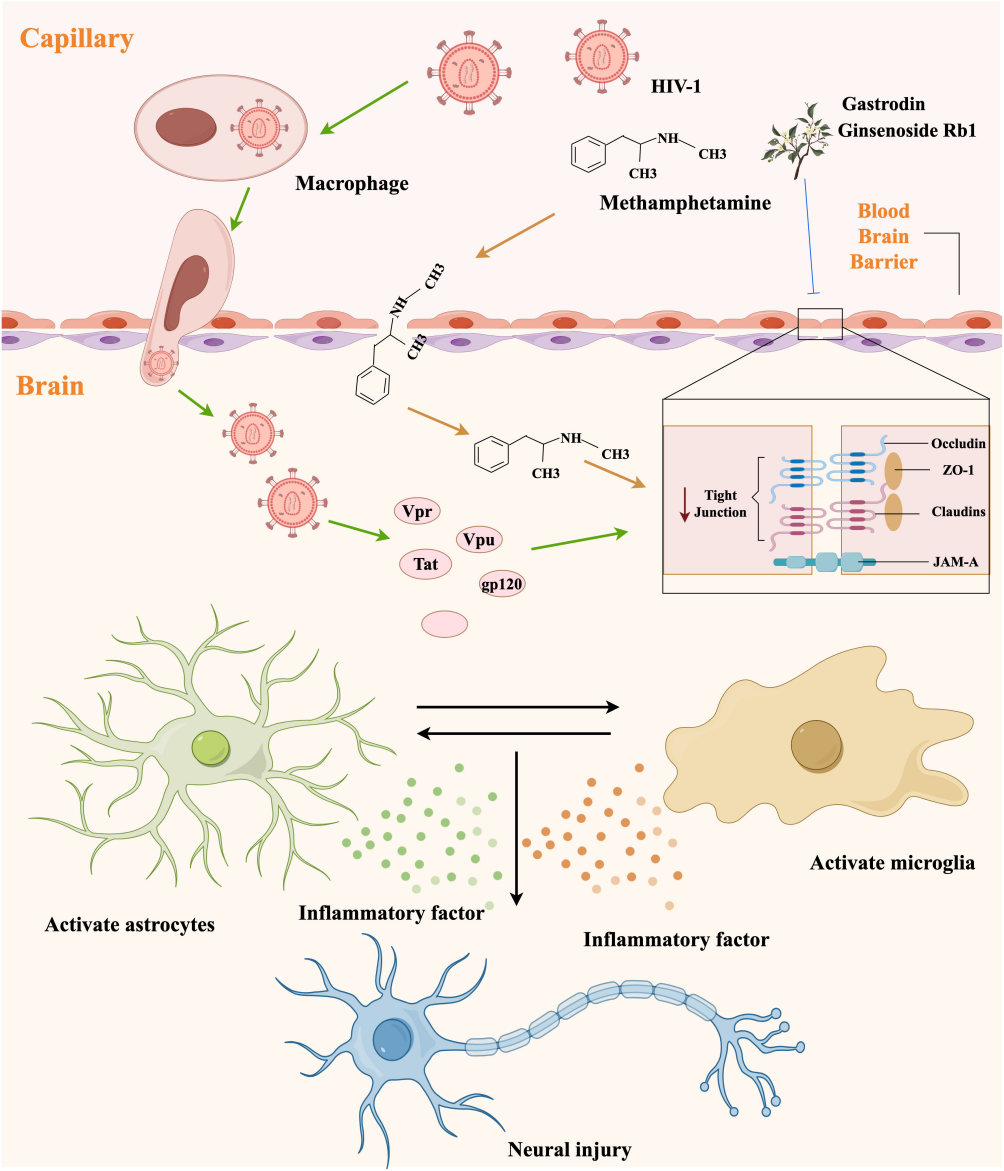
The mechanism diagram of neuroinflammation and blood-brain barrier damage caused by METH and HIV-1.

Disruption of BBB results in infiltration of various plasma components, and immune factors into the CNS, thereby inducing neuroinflammation and ultimately leading to toxic damage to the nervous system (80). The mechanisms of neuroinflammation and BBB damage caused by METH and HIV-1 are summarized in **Figure 3**. METH affects CNS homeostasis by impairing pericytes and astrocytes and disrupting tight junction proteins, such as occludin and claudins (81). Recent research has demonstrated that METH exposure induced neuroinflammation and BBB damage through neuronal-derived alpha-nucleoprotein transfer to astrocytes, as evidenced by the co-culturing of neurons with astrocytes (76). Upon entry into the BBB, HIV-1-infected cells secrete a range of HIV-1-associated viral proteins, including Tat, gp20, and Vpr. Notably, the HIV-1-associated viral proteins can exert disruptive effects on the BBB through various mechanisms (82), such as specifically enhancing vascular permeability, suppressing the expression of tight junction proteins, inducing a reduction in the expression of claudin-5 and laminin, and stimulating the expression of matrix metallopeptidase 9 (MMP9) and matrix metallopeptidase 2 (MMP2), ultimately resulting in the cleavage of tight junction proteins (83, 84). However, the mechanism of injury associated with HIV-1 Tat protein-induced BBB damage is multifaceted and not isolated. Despite extensive research, complete elucidation of the mechanism of injury remains elusive.

In our previous series of experiments, we found that ginsenoside Rb1 and gastrodin could alleviate the synergistically induced BBB damage by METH and HIV-1 Tat protein to a certain extent (85, 86). However, the neurotoxic effects caused by METH and HIV-1 are likely to arise from the interaction of various cellular and molecular mechanisms, and more in-depth studies, as well as additional clinical testing, may be required to achieve a therapeutic cure (22). Furthermore, the BBB presents significant challenges for future pharmacological interventions. Consequently, enhancing the ability of drugs across the BBB remains a prominent research focus and obstacle.

## 3 Potential therapeutic targets for neurotoxicity co-induced by METH and HIV-1

**Figure 4:**
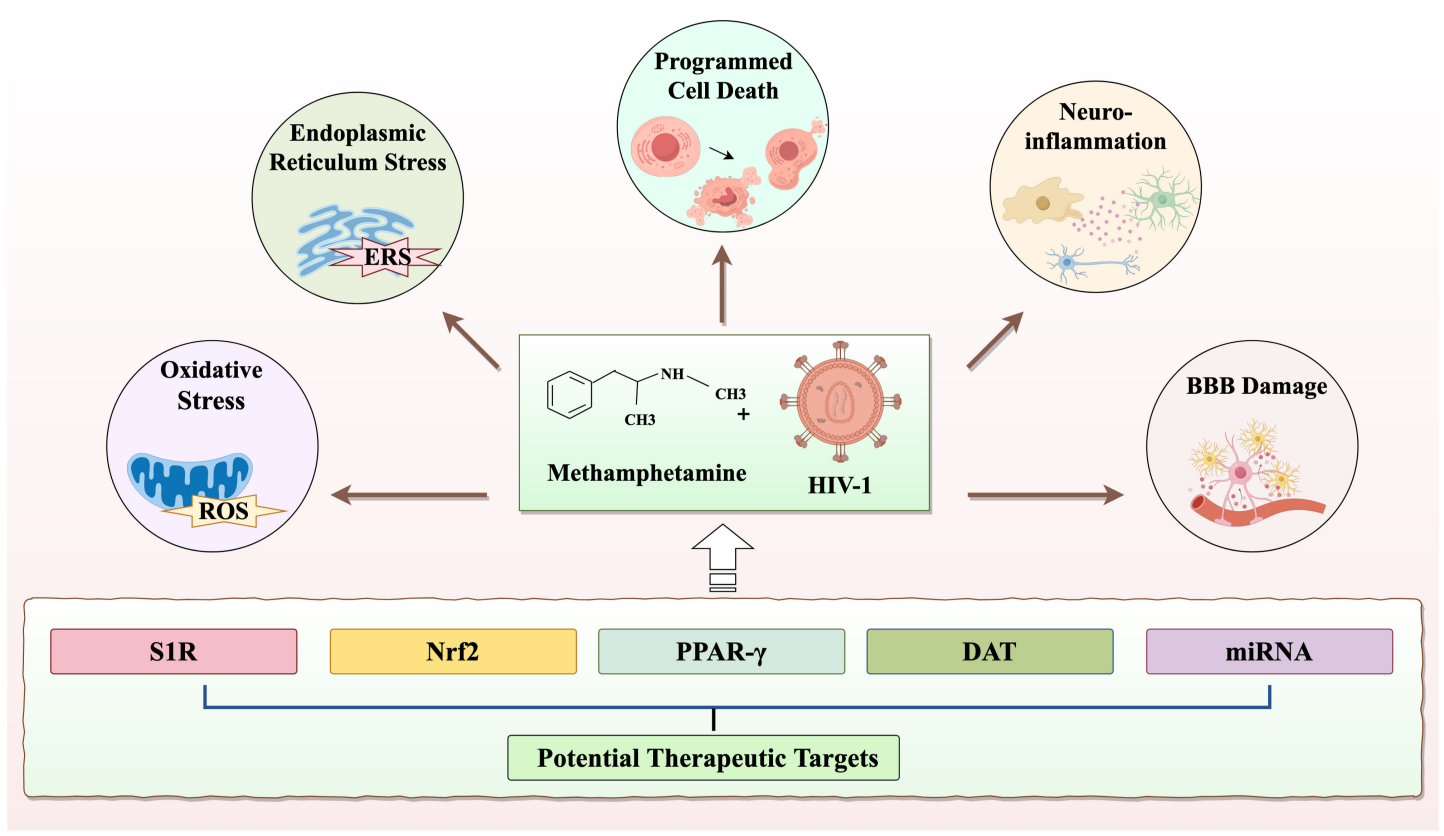
The neurotoxicity and therapeutic targets co-induced by METH and HIV-1.

Despite the significant extension of life expectancy for individuals with HIV-1 through combination antiretroviral therapy (cART), chronic infections persist in the brain, and HIV-1-related neurocognitive impairment remains a formidable, incurable challenge (87). To date, the FDA has not approved any effective treatments for METH-induced neurotoxicity (88). Nevertheless, researchers continue to explore potential therapeutic agents for the neurotoxic effects induced by METH and HIV-1. The potential drugs that may treat METH and HIV-1-induced neurotoxicity are summarized in **Table 1**. For instance, ginsenoside Rb1 has shown promise in mitigating METH-induced neurotoxicity through the NR2B/ERK/CREB/BDNF pathway (89). Gastrodin, another agent, has demonstrated to attenuate METH-induced autophagy and apoptosis (41, 90). However, these agents have not yet provided a complete solution to the problem. Therefore, in this section, we offer a thorough overview of the targets for potential treatment of the neurotoxicity induced by METH and HIV-1 (**Figure 4**).

**Table 1:** The potential drugs that may treat METH and HIV-1-induced neurotoxicity.

Agent	Cell/Animal Type	Curative effect	Mechanisms	Ligand
Ginsenoside Rb1	SH-SY5y cells Rats	Regulated METH-induced neurotoxicity and METH-induced CPP through the NR2B/ERK/CREB/BDNF regulatory pathway. Protect the BBB against the toxic effects of HIV-1 Tat and METH.	Apoptosis BBB damage	METH (89) METH+HIV-1 Tat (86)
Gastrodin	SH-SY5y cells Rat primary cortical neurons Rats Tree shrews Human brain capillary endothelial cells	Enhances the expression of tight junction proteins. Exhibit an anti-autophagic effect on the inhibition of the METH-induced Beclin-1 protein expression, partly via the AKT/mTOR. Regulation of cAMP/PKA/CREB signaling pathway and upregulates the expression of BDNF. Attenuate the effects of METH-induced CPP in rats by regulating the PKA/CREB signaling pathway.	Apoptosis BBB damage Autophagy	METH (41, 90, 91) METH+HIV-1 Tat (85)
MCC950	C57BL/6 mice Rats Primary microglia	Inhibiting NLRP3 alleviated the above-mentioned motor deficits and cerebellar pathologies. Inhibited pyroptosis and release of inflammatory factors.	Neuroinflammation Pyroptosis	METH (68) METH+HIV-1 gp120 (63)
Sigma 1 receptor antagonist	PC12 cells Rats Swiss-Webster mice	Prevents METH-induced sensitization. Mitigate METH-induced mortality and reduce seizures and convulsions. Attenuates conditioned place preference in male rats and viability in PC12 cells through the Sigma1R/AKT/GSK3β/CREB signaling pathway.	Sensitization	METH (92–94)
Sigma 1 receptor agonist	Primary cortical neuronal C57BL/6 mice Sigmar1^-/-^ mice	SCFAs supplementation optimized METH-induced microbial dysbiosis, ameliorated colonic inflammation, and repressed anxiety- and depression-like behaviors. Protecting neurons from gp120 invasion by regulating the expression of bcl-2.	Cognitive disorders Apoptosis	METH (13) HIV-1 gp120 (95)
Nrf2 agonist	BV2 cells SH-SY5y cells Primary microglia Primary hippocampal neurons Nrf2-KO C57BL/6J mice Autopsied brain tissues of METH-abusing and/or HIV-1 infection	Reduced the level of oxidative stress in the organism, thereby attenuating the synergistic induction of autophagy by METH and HIV-1 Tat protein. Nrf2 antagonizes BV2 cell ferroptosis induced by METH and HIV-1 Tat through regulation of SLC7A11. TREs may exert potent neuroprotective effect via activation of both ERK and Nrf2 pathways. Preserves neuronal cells from METH-induced neurotoxicity by upregulating HO-1 expression through the Nrf2 and PI3K/Akt/mTOR signaling pathways. Resveratrol might effectively prevent memory impairment via the interaction with Keap1, activation of the Keap1-Nrf2 pathway, and inhibition of DNA damage and apoptotic responses post METH exposure.	Autophagy Ferroptosis Oxidative stress Apoptosis	METH (96–98) METH+HIV-1 Tat (17, 74)
Chaihu-jia-Longgu-Muli decoction	Rats	Prevent the development of METH-induced withdrawal symptoms in rats	Sensitization Neuroinflammation	METH (99)
DA receptor blocker	C57BL/6 mice	Attenuate METH-induced CPP in mice	Sensitization	METH (100)
DAT mutants	PC12 cells HEK293 cells	Mutating these residues attenuates this inhibitory effect by disrupting the Tat-hDAT interaction	/	HIV-1 Tat (101)
PPAR-γ agonists	Human cerebral microvascular endothelial cells (hCMEC/D3) U937 cells FVB/NJ wild-type or MMP-9-knockout mice Rats C57BL/6 mice BALB/c mice Rat astrocytes and microglia	Prevented the expression of behavioral sensitization to METH challenge on withdrawal day 7. Treatment with ibuprofen, a commonly used nonprescription NSAID, can attenuate METH-induced neurotoxicity and microgliosis. Suppression of the inflammatory response in brain tissue. Against HIV-1-induced MMP-9 expression in brain endothelial cells.	Sensitization Neuroinflammation Oxidative stress BBB damage	METH (102, 103) HIV-1 (104, 105)
Insulin	Autopsied brain tissues, Primary human microglia Primary human neurons Cats(FIV^+^)	Reduced glial cell activation decreased CXCL 10 and IL-6 transcript levels and improved memory and motor function	Neuroinflammation	HIV-1 (106)
miR-204 mimics	HIV-1 transgenic (Tg) rats Wild-type rats The human frontal cortex brain sections Mouse primary glia Adult microglia from the wild-type and HIV-1 Tg rats	Reduced the expression of ACSL4 while inhibiting HIV-1 Tat-mediated ferroptosis and the release of proinflammatory cytokines	Neuroinflammation Ferroptosis	HIV-1 Tat (48)
miRNA-505 inhibitor	Human brain tissues HIV-1 transgenic rats Mouse primary microglial	Upregulated the expression of SIRT3 and mitochondrial antioxidant enzymes, with a concomitant decrease in microglial senescence	Oxidative stress	HIV-1 Tat (105)
Anti-miR-143 lentivirus	Human brain microvascular endothelial cells (HBMECs) C57BL/6 mice PUMA KO mice	Prevented the BBB damage induced by METH	BBB damage	METH (107)
Ibudilast (miR-29a inhibitor)	Macaques Rats The NR‐9460 microglial cell line The NR‐19980 microglial cell line RAW 264.7 macrophages	Attenuated inflammation and rescued synaptodendritic injury	Neuroinflammation	METH (108)
miR-34a inhibitor	TZM-bl cells	Upregulation of protein expression of SIRT1	Neuroinflammation	HIV-1 Tat (29)

### 3.1 Sigma 1 receptor

Initially, Martin et al. proposed that the sigma 1 receptor (σ1R) was a new subtype of opioid receptor that facilitates specific effects induced by benzomorphans (109). Subsequent research has revealed that σ1R, an endoplasmic reticulum chaperone protein, is modulated by various ligands and serves as a potential therapeutic target for psychostimulant addiction (92). The σ1R has multiple functions within the cell, and its activation or inhibition may have different effects on the cell, depending on the specific biological context and pathological conditions (110).

A recent study suggests that supplementation with short-chain fatty acids can improve anxiety and depression-like behaviors induced by METH, through the activation of the σ1R/BDNF/TrkB pathway in the hippocampus of mice (13). Studies have found that HIV-1 gp120 can induce neuronal apoptosis and neuronal generation, while the 4-phenyl-1-(4-phenylbutyl) piperidine (PPBP), σ1R agonist, can weaken the neurotoxicity of gp120 (95). It suggests a protective role for σ1R. However, BD1047, a σ1R antagonist, has been found to mitigate METH-induced mortality and reduce seizures and convulsions in Swiss-Webster mice (93). Prior research also has demonstrated that cannabidiol can attenuate METH-induced conditioned place preference in rats through the σ1R/AKT/GSK-3β/CREB signaling pathway (94). The incidence of HIV-1 infection in quiescent CD4^+^ T cells is typically low; but even minor alterations in these cells can result in increased susceptibility to HIV-1 infection. Similarly, studies have indicated that the utilization of stimulants, such as cocaine, can enhance the likelihood of HIV-1 infection in quiescent CD4^+^ T cells through σ1R activation (111, 112). These findings suggest that σ1R may have damaging effects on the organism. In summary, σ1R plays a complex role in regulating homeostatic processes and may serve as a potential target for future therapeutic interventions.

### 3.2 Transcription factor NF-E2-related factor 2

Nrf2 is a significant transcriptional regulator with anti-inflammatory and antioxidant properties and a safeguarding effect on cellular survival (113). Under normal physiological conditions, Nrf2 is predominantly located in the cytoplasm and associates with Keap1 to form a complex. Upon exposure to stimuli such as ROS, Nrf2 disengages from Keap1 and translocates into the nucleus, where it interacts with antioxidant response elements and triggers the expression of antioxidant enzymes (114). When the extent of damage surpasses its regulatory capacity, the Keap1/Nrf2 pathway can have deleterious effects on the body, exacerbating oxidative stress.

The potential of targeting Nrf2 to treat METH and HIV-1-induced neurotoxicity has been extensively studied. Research has demonstrated that the endogenous antioxidant pathway, mediated by Nrf2, can mitigate METH-induced neurological dysfunction resulting from oxidative stress (96, 97). Resveratrol, a natural polyphenol, has been extensively studied for treating neurodegenerative diseases (115). Numerous studies have shown that multiple exposures to METH significantly impair cognitive function, leading to long-term memory deficits (116), whereas resveratrol may be effective in preventing memory impairment through interaction with Keap1, activation of the Keap1-Nrf2 pathway, and inhibition of DNA damage and apoptotic responses after METH exposure (98). Additionally, Nrf2 has also been reported to regulate iron homeostasis *in vivo (* 117). It has been shown that Nrf2 can respectively prevent ferroptosis and autophagy induced by METH and HIV-1 Tat proteins by regulating the SLC7A11 and Nrf2/NQO1/HO-1 signalling pathways (17, 74). Consequently, further investigation of the functional role of Nrf2 provides great value for the treatment of METH and HIV-1-induced neurotoxicity.

### 3.3 Dopamine transporter protein

DA is crucial for regulating various physiological processes, such as reward, motivation, movement, working memory, and cognition (118). Dopaminergic neurons contain a transmembrane protein, DAT, which primarily promotes reuptake of DA released into the synaptic cleft (119).

Acute or single high dose METH abuse is indicated to cause a decrease in the amount of DAT and affect DA reuptake. This causes an increase in extracellular concentrations of DA, which in turn leads to the euphoria effect (120). However, the reduction in DAT can persist for up to 3 years after METH discontinuation. Prolonged or repeated METH administration can lead to a decrease in DA levels (121). The administration of traditional Chinese medicines, such as Chaihu-jia-Longgu-Muli decoction and levo-tetrahydropalmatine, has been demonstrated to regulate the levels of DA intracellular and extracellular, thereby mitigating the neurotoxicity associated with METH (99, 100). It has been posited that long-term HIV-1 viral protein exposure leads to a decreased dopaminergic state, which continues to persist despite the advent of cART (122).. Therefore, HIV-1 treatment should prioritize the restoration of DA function. The HIV-1 Tat protein plays a crucial role in the onset of HAND by binding to DAT, impeding DA transmission, and inducing neurotoxicity (123). Research has demonstrated that the HIV-1 Tat protein inhibits DA uptake by directly interacting with the human-dopamine transporter protein (hDAT) through mutation of crucial hDAT residues, namely D-H547, D-Y88, and D-D206, which are anticipated to participate in the binding process. This inhibition can be attenuated by disrupting the Tat-hDAT interaction via mutations in these residues (101). In the context of the combined influence of METH and HIV-1, the HIV-1 Tat protein has been observed to intensify the sensitizing impact of METH by altering the DA function (124). These findings indicate that extrasynaptic DA levels and DATs are crucial elements to focus on in managing neurotoxicity caused by METH and HIV-1, potentially paving the way for novel avenues in interventional research.

### 3.4 Peroxisome proliferator-activated receptor gamma

Peroxisome proliferator-activated receptor gamma (PPAR-γ) belongs to the nuclear hormone receptor superfamily of ligand-activated transcription factors that govern various functions, such as lipid metabolism and inflammation (125). Additionally, PPAR-γ plays a crucial anti-inflammatory role in brain injury and neurodegenerative diseases, is implicated in the pathogenesis of several diseases, including diabetes, cancer, and obesity, and is a promising therapeutic target for CNS disorders (126).

Activation of PPAR-γ results in its translocation to the nucleus, increasing anti-inflammatory gene expression and decreasing microglia/macrophage activation, ultimately downregulating proinflammatory factors (127). Behavioral sensitization, an experimental model of psychostimulant psychosis, is induced by repeated administrations of psychostimulants and has linked to brain inflammation. Notably, PPAR-γ activation attenuated METH-induced behavioral sensitization, potentially due to its anti-inflammatory properties (102). After the three-day METH injection, a gradual decrease in PPAR-γ expression was observed. However, subsequent treatments with aspirin and ibuprofen gradually reversed the DAT expression reduction, microglial activation, and PPAR-γ expression. These findings suggest a correlation between the reduction of PPAR-γ and METH-induced neurotoxicity, implying that the PPAR-γ agonistic effect of ibuprofen may underlie its ability to reverse neurotoxicity (103).

HIV-1 infection results in a range of neurological dysfunctions that entail activating glial cells and releasing inflammatory factors. These events lead to the occurrence of lethal toxic effects on nerve cells (104). Insulin treatment has been observed to upregulate PPAR-γ expression in HIV-1-infected primary microglia (106). Furthermore, HIV-1 primarily infects the CNS by disrupting the BBB. A reduction in PPAR-γ impedes MMP-9 and MMP-2 activity in human monocytes infected with HIV-1, leading to BBB impairment (105). Inflammation is a common factor in both METH and HIV-1 induced neurotoxicity, and the anti-inflammatory properties of PPAR-γ provide compelling evidence for synergistic therapeutic investigation of these conditions.

### 3.5 miRNA

The emergence of diverse genetic testing technologies, including transcriptome sequencing, has increased the interest in the *in vivo* function of non-coding RNAs (128), including rRNA, tRNA, snRNA, snoRNA, and microRNA. Recent extensive research has established that miRNAs can contribute to disease pathogenesis through various mechanisms and serve as viable targets for therapeutic drugs (129). This section provides a concise summary of the involvement of miRNAs in METH and HIV-1-induced neurotoxicity.

The pivotal function of miRNAs is contingent on their target genes, as established in the literature. Extensive studies have supported an important role for miRNAs in neurotoxicity induced by METH and HIV-1 (48, 130, 131). Specifically, METH induces the upregulation of miRNA-143 via σ1R, resulting in detrimental effects on human brain endothelial cells and disruption of tight junction proteins, ultimately compromising BBB integrity. Notably, silencing of miRNA-143 has been shown to confer protection to the BBB against METH-induced damage (107). Studies have demonstrated that chronic administration of METH leads to an increase in the expression of exosome-secreted miRNA-29a. However, ibudilast, an anti-inflammatory drug, can reduce the secretion of extracellular vesicles and subsequently downregulate miRNA-29a, mitigating synaptic damage (108). HIV-1 neurotoxicity is closely linked to its infectious capacity, and various stimuli can alter miRNA expression *in vivo*, thereby influencing HIV-1 replication. Activated CD4+ T cells can exhibit high levels of miRNA-132, the first miRNA discovered to enhance HIV-1 replication (132). Furthermore, the elevation of miR-34a by HIV-1 Tat protein impeded SIRT1, consequently triggering neuroinflammation (29). These results highlight the importance of miRNA-based gene therapy in addressing the neurotoxicity of both METH and HIV-1, as these miRNAs have been shown to be stably expressed *in vivo*.

## 4 Discussion

The excitotoxicity induced by METH leads to an elevated likelihood of engaging in risky sexual behavior and unhygienic injection practices among substance abusers, thereby increasing the risk of HIV-1 acquisition. This risk is further exacerbated in individuals with co-infection of HIV-1. Currently, there are no pharmacological interventions specifically targeting METH-induced neurotoxicity, with only symptomatic treatments available based on clinical presentation. Antiretroviral therapy can effectively manage HIV-1, but it does not completely remove the virus from the brain. In addition, a variety of complementary therapies can be employed, including psychotherapy, lifestyle changes, and social support. The above treatments primarily relieve clinical symptoms without preventing disease progression. Therefore, researchers are trying to find effective drugs that can treat the neurotoxicity they co-cause by exploring the common targets of METH and HIV-1. In this review, we have provided a comprehensive and up-to-date overview of the mechanisms and targets currently under investigation for METH and HIV-1-induced neurotoxicity, drawing on published literature and recent reports.

We have identified that σ1R, NRF2, DAT and PPAR-γ are all involved in METH and HIV-1 co-induced neurotoxicity (13, 17, 125, 133). Many preclinical studies have suggested that modulation of these targets may have significant therapeutic effects on METH and HIV-1 co-induced neurotoxicity. This provides new perspectives and potential breakthroughs for such future therapeutic strategies. However, future research should encompass more in-depth experiments and foster multidisciplinary communication to identify precise biomarkers. These biomarkers are instrumental for the early detection and classification of METH and HIV-1 co-induced neurotoxicity, thereby facilitating the development of personalized treatment plans. Meanwhile, we also found that METH and HIV-1 co-induced neurotoxicity has been significantly altered at the transcriptional level, and we briefly summarized some aberrant miRNAs, such as miRNA-143, miRNA-29a and miRNA-132 (108, 131, 134). The researchers have found that targeting these miRNAs could also ameliorate METH and HIV-1 co-induced neurotoxicity, providing a new strategy for treatment. However, the clinical application of miRNA therapy continues to face a lot of challenges, including issues of drug delivery, specificity, and safety, which need to be addressed through further research and clinical trials.

We believe that there are numerous unanswered questions for researchers to explore regarding the neurotoxicity associated with METH and HIV-1 co-exposure. Specific questions include: I. How METH affects HIV-1 latency and reactivation. II. The role of environmental factors in exacerbating or mitigating the neurotoxicity. III. The effects of simultaneous use of different drugs on the co-induced neurotoxicity of METH and HIV-1. IV. The specific roles of cellular populations and neural circuits in the neurotoxicity induced by the co-exposure to METH and HIV-1. Meanwhile, we should not overlook the impact of the social environment on patients. We must enhance public awareness of substance use disorder and HIV-1 infection through education and prevent such issues at their root. It is crucial to provide emotional support and practical assistance to individuals affected by HIV-1 and METH use, to promote societal understanding and acceptance of them, thereby reducing their psychological barriers to seeking help and improving their quality of life. In conclusion, we hope more researchers will focus on exploring treatments for METH and HIV-1-induced neurotoxicity.

## 5 Conclusion

The review provided an overview of the distinct mechanisms underlying neurotoxicity induced by METH and HIV-1, emphasizing their interplay. These mechanisms include oxidative stress, endoplasmic reticulum stress, neuroinflammation, programmed cell death, and blood-brain barrier damage. Additionally, potential targets for treating METH and HIV-1-induced neurotoxicity were discussed. The objective of this review was to establish a theoretical foundation for understanding METH and HIV-1-induced neurotoxicity and to suggest potential avenues for clinical research and treatment.
